# *In silico* Evaluation of PLAC1-fliC As a Chimeric Vaccine against Breast Cancer

**DOI:** 10.29252/ibj.24.3.173

**Published:** 2019-11-18

**Authors:** Mortaza Taheri-Anganeh, Ahmad Amiri, Ahmad Movahedpour, Seyyed Hossein Khatami, Younes Ghasemi, Amir Savardashtaki, Zohreh Mostafavi-Pour

**Affiliations:** 1 Department of Medical Biotechnology, School of Advanced Medical Sciences and Technologies, Shiraz University of Medical Sciences, Shiraz, Iran;; 2 Department of Biochemistry, School of Medicine, Shiraz University of Medical Sciences, Shiraz, Iran;; 3 Department of Pharmaceutical Biotechnology, School of Pharmacy, Shiraz University of Medical Sciences, Shiraz, Iran;; 4 Pharmaceutical Sciences Research Center, Shiraz University of Medical Sciences, Shiraz, Iran; 5 Maternal-Fetal Medicine Research Center, Shiraz University of Medical Sciences, Shiraz, Iran

**Keywords:** Bioinformatics, Breast cancer, Cancer vaccines, PLAC1

## Abstract

**Background:**

Breast cancer is one of the most prevalent cancers among women. Common cancer treatment methods are not effective enough, and there is a need for a more efficient treatment procedure. Cancer vaccine is a novel immunotherapy method that stimulates humoral and/or cellular immunity against cancer. PLAC1 is a cancer/testis antigen, prevalent in breast cancer and rarely found in normal tissues. FliC, as a bacterial adjuvant, when fused to PLAC1 can elicit humoral and cellular responses. Therefore, PLAC1-fliC is a chimeric protein, which can be considered a suitable candidate against breast cancer.

**Methods:**

ProtParam was used to evaluate the physicochemical properties of PLAC1-fliC. Second structures were determined using the GOR V server. PLAC1-fliC 3D structure was modeled by Phyre2, and it was refined using GalaxyWEB. The refined model was submitted to RAMPAGE, PROCHECK, and ProSA-web for validation. Antigenicity and allergenicity of the construct were predicted by ANTIGENpro, VaxiJen, AllergenFP, and SDAP databases. Then MHC-I- and MHC-II-binding epitopes of PLAC1-fliC were forecasted by NetMHC 4.0 and NetMHCII 2.3 Servers. Finally, Ellipro and CTLpred were employed to predict B-cell and CTL epitopes.

**Results:**

The construct was evaluated as a stable fusion protein, which could be antigenic and could stimulate B and T cells against breast cancer.

**Conclusion:**

PLAC1-fliC, as a cancer vaccine candidate, might be suitable and specific for breast cancer, which could evoke humoral and cellular immunity against this type of tumor.

## INTRODUCTION

Breast cancer is the most frequently diagnosed cancer and cancer-related death amongst women. Annually, more than two million breast cancer cases are reported worldwide^[^^1^^]^. Despite the conventional therapeutic modalities for breast cancer treatment, these therapies have been suggested to be rather ineffective^[^^2^^]^.

New studies have shown that immunotherapy can be an effective and alternative choice in treating breast cancer^[^^3^^]^. Immunotherapy consists of anti-cancer antibody, cancer vaccine, and T-cell receptor engineering^[^^2^^]^. PLAC1 is a cancer/testis antigen with a significant role in cancer progression and invasion^[^^4^^]^. Cancer/testis antigens are specific antigens expressed in germ-line cells like testis, fetal ovary, and placenta. These antigens are also expressed in some cancer cells, but rarely found in normal tissues^[^^5^^]^. Studies have revealed that PLAC1 expression in normal cells is not adequate to be measured, while its expression in cancer cells is measurable^[^^5^^,^^6^^]^. Recently, it has been displayed that PLAC1 plays a critical role in tumor invasion and metastasis through Furin/NICD/PTEN/AKT axis^[^^7^^]^. PLAC1 is a type II membrane bound protein, which its 5-22 amino acids form a transmembrane helix, and has a large extracellular domain consisting of 23-212 amino acids. A truncated zona pellucida domain in the extracellular part of PLAC1 is made of amino acids 29-119^[^^8^^]^. New research on PLAC1 has indicated that it is well expressed in a versatile cancer cases, specifically in breast cancer, but not expressed in normal tissues, except testis^[^^9^^]^. Therefore, PLAC1 could be a specific target for breast cancer immunotherapy. 

 Vaccines need an adjuvant to be more effective^[^^10^^]^. Adjuvants induce TLR, which results in helper T-cell activation. Bacterial flagellin is one of the most important protein adjuvants that induces TLR5 receptors^[^^11^^]^. FliC protein is the main component of flagellin and encoded by *flic* gene^[^^12^^]^. *Salmonella enterica* serovar typhimurium FliC, as an efficient adjuvant, is widely used in vaccine research^[^^13^^]^. FliC is made of four domains, including D0, D1, D2, and D3. D1 is responsible for TLR5 binding and dimerization of TLR5s and triggers the downstream signaling and stimulates cells to secrete proinflammatory cytokines such as TNF-α^[^^14^^]^. Flagellin is a TLR5 binding ligand and starts downstream signaling through MyD88 pathway, which activates innate immunity. It has been shown that the innate immune system motivation results in cytokines secretion and dendritic cells activation^[^^15^^]^. 

The aim of this study was to design a fusion protein construct, as an effective vaccine, consisting of PLAC1 (as a specific antigen) and *Salmonella enterica *fliC (as a bacterial adjuvant) that can stimulate humoral and cellular immune responses against breast cancer. This construct was evaluated using bioinformatics online web servers. 

## MATERIALS AND METHODS

**Construct design**

In this study, the amino acid sequences of PLAC1 and fliC were extracted from Uniprot database (https://www.uniprot.org/) in FASTA format (Uniprot id: Q9HBJ0). Amino acids 23-212 of PLAC1 were considered for the construct design, and residues 1-22 were neglected because they are located in plasma membrane and cytosol, and the humoral immunity does not have access to them. A flexible linker (GSGGSGGSGGSG) was located between PLAC1 antigen and fliC adjuvant. Our final construct was PLAC1 (23-212)-linker (GSGGSGGSGGSG)-fliC. 

**Prediction of physicochemical properties and** **secondary structure **

To predict different physicochemical features, such as instability index, isoelectric point, aliphatic index, grand average of hydropathicity, and molecular weight for PLAC1-fliC, we utilized ProtParam server (https://web.expasy.org/protparam/)^[^^16^^]^. Secondary structure of PLAC1, fliC, and PLAC1-fliC were predicted using GOR V server (https://abs.cit.nih.gov/ gor/) and were compared together^[^^17^^]^.

**Tertiary structure prediction and refinement**

Phyre2 server (http://www.sbg.bio.ic.ac.uk/~phyre2/ html/page.cgi?id=index) was employed to predict the 3D structure of the construct based on homology modeling method^[^^18^^]^. The model was refined using GalaxyRefine server (http://galaxy. seoklab.org/cgi-bin/submit.cgi?type=REFINE)^[^^19^^]^, and the best refined model was selected and submitted for next steps.

**Validation of tertiary structure**

For the validation of the model, the following servers were used: RAMPAGE (http://servicesn.mbi.ucla.edu/ PROCHECK/), ProSA-web (https://prosa.services. came.sbg.ac.at/prosa.php), and PROCHECK (http:// servicesn.mbi.ucla.edu/PROCHECK/). RAMPAGE server has ability to check stereochemical qualities of the models peptide bonds and shows the number of residues in favored, allowed and outer areas in a Ramachandran plot. ProSA-web has a diagnostic method that is able to analyze protein structures based on all the available protein structures^[20]^. PROCHECK server was employed for evaluating the stereochemical quality of the PLAC1-fliC^[^^21^^]^. The results of all the three servers were compared before and after 3D structure model refinement.

**Antigenicity** **and allergenicity prediction**

VaxiJen server (http://www.ddg-pharmfac.net/ vaxijen/VaxiJen/ VaxiJen.html) was used for the prediction of protective antigens and subunit vaccines. According to the physicochemical properties of proteins, this server classifies antigens without referral to sequence alignment. The accuracy of the server based on the origin of the protein (bacterial, viral, and tumor protein datasets) varies between 70 and 89%^[^^22^^]^. Antigenicity of the construct was rechecked by ANTIGENpro (http://scratch.proteomics.ics.uci.edu/), which is based on pathogen independent, sequence-based, alignment-free analysis and uses antigenicity microarray data for predicting the protein antigenicity. SDAP (http://fermi.utmb.edu/SDAP/sdap_man.html) and AllergenFP (http://www.ddgpharmfac.net/ AllergenFP/) databases were employed for allergenicity prediction of the fusion protein. SDAP is the structural database of allergenic proteins, while AllergenFP online bioinformatics tool is based on descriptor fingerprint^[^^23^^]^.

**Fig. 1:**
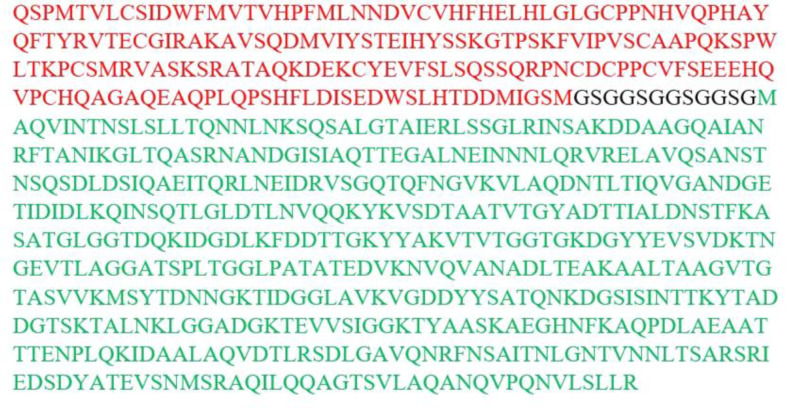
Amino acid composition of PLAC1-fliC construct. Red shows PLAC1, black indicates a flexible linker, and green shows fliC

**MHC-I and MHC-II binding epitope prediction**

NetMHC 4.0 Server (http://www.cbs.dtu.dk/services/ NetMHC/) and NetMHCII 2.3 Server (http://www.cbs. dtu.dk/services/NetMHCII/) were applied to predict MHC-I binding epitopes (based on an artificial neural networks method) and MHC-II binding epitopes, respectively^[^^24^^,^^25^^]^. 

**B cell and CTL epitopes prediction**

B-cell epitopes, both continuous and discontinuous, were predicted using ElliPro server (http://tools. iedb.org/ellipro/)^[^^26^^]^. CTLPred server (http://crdd.osdd. net/raghava/ctlpred/) was utilized for the prediction of CTL epitopes based on the direct method. This method uses data on T-cells epitopes templates instead of MHC-binding peptides. CTLpred method is based on techniques such as artificial neural network and support vector^[^^27^^]^. 

## RESULTS

**Physicochemical properties**

The fusion construct was designed (Fig. 1). The predicted molecular weight and isoelectric point for our fusion protein were 73642.54 Da and 5.17, respectively. Total numbers of negatively and positively charged amino acids of this protein were 73 and 55; thus, its final charge was -18, and it totally had a negative charge. Instability index for PLAC1-fliC protein was 32.87, which was considered as the stable protein; the instability index under 40 means stability. Aliphatic index and grand average of hydropathicity of our fusion protein were 76.85 and -0.380, respectively.

**Secondary structure**

**Fig. 2:**
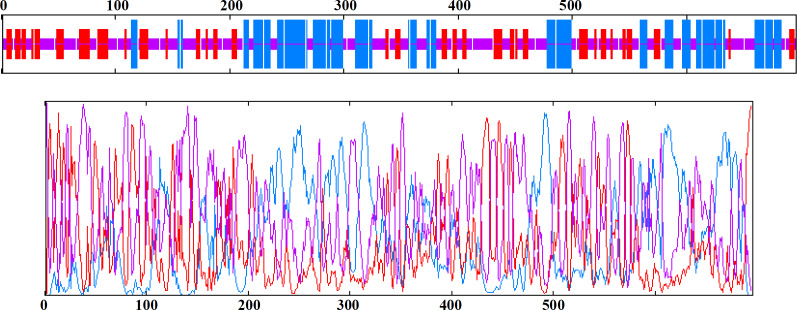
Graphical results of secondary structures comparison. Helix, extended strand, and random coil are indicated by blue, purple and red, respectively

Our findings showed that PLAC1-fliC had 29.31% alpha helix, 21.98% extended strand, and 48.71% random coil. Comparison between PLAC1, fliC, and PLAC1-fliC is shown in Table 1, and the secondary structure pattern of PLAC1-fliC is indicated in Figure 2.

**Homology model building, refinement, and validation**

**Fig. 3:**
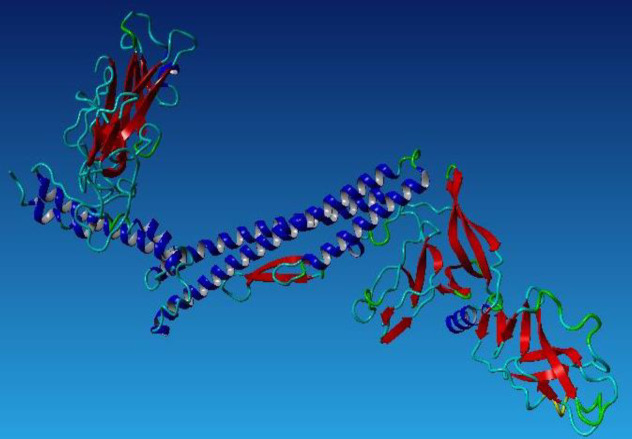
The 3D structure of PLAC1-fliC defined by Phyre 2.

**Fig. 4.**
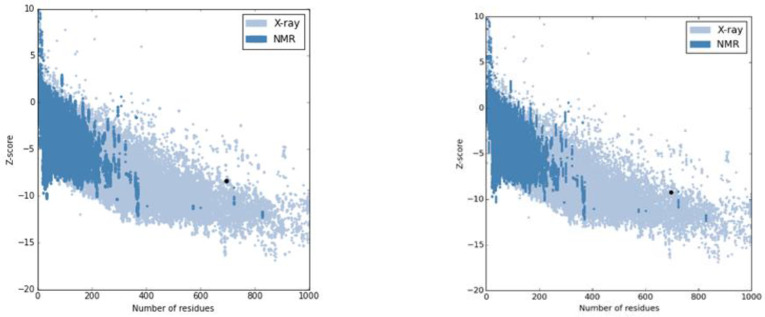
The Z-score plot for the 3D model of PLAC1-fliC before (A) and after (B) refinement. Z-score is shown by a black spot

The PLAC1-fliC protein model was built using Phyre2 based on Hidden Markov Model. Figure 3 depicts fusion protein predicted tertiary structure. This model was refined using GalaxyRefine server, and five refined models were built, and the best one was chosen. ProSA Z-scores for PLAC1-fliC 3D model after and before refinement were -8.38 and -9.21, respectively (Fig. 4). RAMPAGE results showed that the numbers of residues increased in the favored region after the model refinement. These findings were confirmed by PROCHECK results (Table 2). 

**Table 1:** Comparison of PLAC1, fliC, and PLAC1-fliC fusion secondary structures

**Protein**	**Alpha helix (%)**	**Extended strand (%)**	**Random coil (%)**
PLAC1	5.26	36.32	58.42
fliC	39.27	16.19	44.53
PLAC1-fliC	29.31	21.98	48.71

**Antigenicity and allergenicity**

ANTIGENpro and VaxiJen results revealed that fliC, as an adjuvant, could increase the antigenicity of PLAC1 protein. Table 3 shows the antigenicity of PLAC1, fliC, linker, and PLAC1-fliC fusion protein. In accordance with AllergenFP server, our findings suggested that PLAC1, fliC, and PLAC1-fliC could not be an allergen for human body. Searching PLAC1, fliC, and PLAC1-fliC sequences in SDAP database showed that these sequences were not allergens, and, therefore, AllergenFP results were confirmed.

**MHCI and MHCII-binding epitopes**

Since 9-mer peptides display more tendency to HLA1, peptides with nine amino acids were chosen. The 9-mer peptides with strong binding preference to HLA-A0201 subtype with their affinity and rank are shown in Table 4. To predict MHC-II binding, 15-mer peptides binding to DRB1_ 0301, DRB1_ 0401, DRB1_ 0701, and DRB1_ 1501 were evaluated. Our finding exhibited that PLAC1-fliC epitopes were strong binders to DRB1_ 0301 and DRB1_ 0401 (Table 5).

**B cell and** **CTL** **epitopes**

Using ElliPro, B cell specific continuous (linear) and discontinuous (conformational) epitopes were defined. Accordingly, 16 continuous epitopes were identified, and their length was between 6-110 amino acids (Table 6). In addition, 4 discontinuous epitopes were predicted with maximum 177 and minimum 15 amino acids in length (Table 7). Top 10 CTL peptide epitopes with higher scores predicted by combined approach, artificial neural network/support vector machine, are shown in Table 8.

## DISCUSSION

According to the global cancer statistics in 2018, breast cancer is the most commonly diagnosed and the leading cause of cancer-related death among women^[^^1^^]^. However, there are merely few therapeutic strategies with limited success against breast cancer^[^^28^^]^. One new therapeutic strategy against cancer is immunotherapy. Cancer vaccine is one of the most effective immunotherapy treatments, but its design and production is often time-consuming and costly. The new generation of vaccines are based on recombinant protein technology according to their specific design, safety, purity, and feasibility of production^[^^29^^]^. Thus, finding and screening the putative vaccine candidate is a prerequisite for its production. In a previous study, Kawada *et al.*^[^^30^^]^ vaccinated 13 patients using NY-ESO-1, a cancer-testis antigen. This antigen induces antibody production, as well as CD4 and CD8 T-cell responses. Furthermore, Shim *et al.*^[^^31^^]^ showed that cancer-associated peptides, as a cancer/testis antigen, can stimulate CTL. Thanks to the integration of biology and computer sciences, there are many available software and webservers that enable us to design and validate biological processes in a dry lab as *in silico*. Hence, we designed a candidate vaccine as a fusion protein, containing PLAC1, which is expressed highly in several types of tumor tissues, but not in normal ones, except placenta and testis^[^^32^^]^. PLAC1 is a suitable candidate for cancer immunotherapy, since it is overexpressed in more than 80% of breast cancers samples, while overexpression of Her2/neu, the target of Herceptin monoclonal antibody, is utmost 25%^[^^8^^,^^33^^]^. Second part of this fusion protein vaccine is ﬂiC from *Salmonella enterica*, which as an adjuvant molecule might be able to stimulate both humoral and cellular immune responses^[^^34^^]^. Third part of this construct is a flexible linker, GSGGSGGSGGSG, which resides between the mentioned parts and contains repeated small or polar amino acids (glycine and serine). Such linkers provide favorable solubility and flexibility and join two domains of fusion protein without changing their structure and function^[^^35^^,^^36^^]^.

 The construct structure was analyzed and validated using diﬀerent bioinformatics tools and servers. Based on the results of different physicochemical parameters and structural analysis, PLAC1-fliC is a stable hydrophilic protein with a net negative charge. Its favorite degree of stability enables it to circulate easily and to be processed by antigen-presenting cells because proteins with a very loose conformation are susceptible to tertiary conformational epitope changes and might degrade easily. On the other hand, rigid structures might resist against antigen processing and epitope presentation^[^^37^^]^.

Secondary structures of polypeptides play an important role in their ultimate structure and function. Forecasting secondary structure from their amino acids sequences is the prerequisite for proteins 3D structure prediction, as well as for prediction of protein stability. GOR V server was used to predict the secondary structure of PLAC1-fliC. It works according to Garnier-Osguthorpe-Robson method that uses combined mathematical tools, probability methods, and data have been extracted from empirical methods like NMR and crystallography^[^^38^^]^. Table 1 reveals that the percentage of PLAC1-fliC random coil and alpha helix reduced and ioncreased, respectively. Therefore, its structure got more regular in comparison with PLAC1. Indeed, alpha helices are more stable than beta strands and random coils and more firm to conformational changes^[^^39^^]^. PLAC1-fliC modeling, refinement and validation were accomplished via different servers. Based on RAMPAGE results, most residues were located in allowed and favorite regions, and even the scores got better after refinement. These results were validated by PROCHECK, as well (Table 2). The predicted model of PLAC1-fliC, when compared with the experimental structures, indicated that this model was valid and matched with experimentally determined structures. Appropriate ProSA Z-scores confirms the accuracy of this model (Fig. 4)^[^^20^^]^.

**Table 2:** Comparison of residues residence in Ramachandran plot before and after model refinement

**Database**	**Before refinement **	**After refinement**
RAMPAGE		
Number of residues in		
Favored region (%)	603 (86.9)	640 (92.2)
Allowed region (%)	54 (7.8)	33 (4.8)
Outlier region (%)	37 (5.3)	21 (3.0)
		
PROCHECK		
Residues in		
Most favored regions (%)	500 (81.2)	528 (85.7)
Additional allowed regions (%)	81 (13.1)	63 (10.2)
Generously allowed regions (%)	23 (3.7)	14 (2.3)
Disallowed regions (%)	12 (1.9)	11 (1.8)

**Table 3:** Antigenicity predictions of PLAC1, fliC, linker, and PLAC1-fliC

**Protein**	**ANTIGENpro**	**VaxiJen** ^*^
PLAC1	0.947422	0.6587
fliC	0.923670	0.8246
linker	0.453669	1.8025
PLAC1-fliC	0.955086	0.7320

* Threshold = 0.5

Since the presence of flagella protein domain, as an adjuvant in the construct, might be allergenic for human; it is necessary to evaluate its potential allergenicity. It is important that vaccines could not be an allergen for humans. Allergens provoke hosts immune system and might lead to undesirable allergenic reactions^[^^40^^]^. The allergenicity prediction result showed that PLAC1-fliC was not allergen for humans; therefore, it can be administrated safely.

For stimulating immune responses against cancer cells and eventually eradicating them, their specific antigen epitopes have to be presented to immune cells, including B and T lymphocytes. Intrinsic antigens like cancer epitopes are presented to CTL by MHC-I, and extrinsic antigen such as adjuvants are presented to T helper cells by MHC-II molecules. Commonly, MHC-I presents 8-11 amino acids of peptides, while MHC-II presents peptides with 12-25 amino acids in length; they interact with T-cell receptor and eventually lead to cellular immune response^[^^41^^]^. The MHC genes are one of the most polymorphic human genes, and their frequency varies among populations, races, and ethnic groups. To predict the presenting potency and binding affinity of PLAC1-fliC in antigen-presenting cells, we selected the MHC-I and MHC-II alleles based on their global and regional relative haplotypes frequency according to scientific reports by investigating their frequency in allele frequency database^[^^42^^-^^46^^]^. As Table 4 and 5 show, immunodominant 9-mer peptide of PLAC1-fliC strongly binds to MHC-I haplotype HLA-A0201, and 15-mer epitope of PLAC1-fliC binds to DRB1_ 0301 and DRB1_ 0401 MHC-II haplotypes. These results exhibit that PLAC1-fliC could be presented efficiently to immune cells and could evoke immunity against cancer cells. In addition, circulating antigens like protein-based vaccines could act as B-cell epitope and result in B-cell activation, antibody production, and humoral immunity stimulation. B-cell epitope is an antigenic determinant, recognized by antibodies or B-cell receptors. It can be a peptide, called the linear or continuous epitope, or it can have a 3D structure which is considered as a conformational or discontinuous epitope. B-cell epitopes of an antigen are antibody production stimulators^[^^26^^]^. Based on Tables 6-8, PLAC1-fliC has both linear and conformational epitopes with strong affinity to B and T cells; therefore, it could potentially evoke humoral and cellular immunity.

In conclusion, our investigation regarding probable vaccine for breast cancer reveals that PLAC1-fliC contains suitable structure and stability that could effectively stimulate both cellular and humoral immunity and might be safe to administer. Since our study is a bioinformatics analysis, experimental studies are warranted for validity of our results.

**Table 4:** HLA1-binding peptides based on NetMHC 4.

**Peptide sequence**	**Position**	**HLA**	**Affinity (nM)**	**Rank (%)**	**Binding level**
FMLNNDVCV	19	HLA-A0201	7.53	0.07	SB
FMVTVHPFM	12	HLA-A0201	10.81	0.12	SB
SIDWFMVTV	8	HLA-A0201	26.82	0.40	SB
VLCSIDWFM	5	HLA-A0201	35.00	0.50	SB

SB, strong binder

**Table 5:** HLA2-binding peptides based on NetMHCII 2.3

**Peptide sequence**	**Position**	**HLA**	**Affinity (nM)**	**Rank (%)**	**Binding level**
DTTIALDNSTFKASA	1	DRB1_0301	15.5	0.50	SB*
ADTTIALDNSTFKAS	2	DRB1_0301	17.0	0.50	SB
TTIALDNSTFKASAT	3	DRB1_0301	17.3	0.60	SB
YADTTIALDNSTFKA	4	DRB1_0301	22.0	0.80	SB
HPFMLNNDVCVHFHE	5	DRB1_0301	22.7	0.80	SB
PFMLNNDVCVHFHEL	6	DRB1_0301	24.3	0.90	SB
VHPFMLNNDVCVHFH	7	DRB1_0301	27.2	1.10	SB
TIALDNSTFKASATG	8	DRB1_0301	33.6	1.40	SB
FMLNNDVCVHFHELH	9	DRB1_0301	40.2	1.70	SB
GYADTTIALDNSTFK	10	DRB1_0301	42.3	1.80	SB
QNRFNSAITNLGNTV	1	DRB1_0401	6.3	0.01	SB
VQNRFNSAITNLGNT	2	DRB1_0401	6.6	0.01	SB
NRFNSAITNLGNTVN	3	DRB1_0401	7.3	0.03	SB
AVQNRFNSAITNLGN	4	DRB1_0401	8.1	0.04	SB
RFNSAITNLGNTVNN	5	DRB1_0401	11.7	0.12	SB
GAVQNRFNSAITNLG	6	DRB1_0401	16.1	0.30	SB
DSDYATEVSNMSRAQ	7	DRB1_0401	17.7	0.40	SB
SDYATEVSNMSRAQI	8	DRB1_0401	19.0	0.40	SB
EDSDYATEVSNMSRA	9	DRB1_0401	19.7	0.50	SB
IEDSDYATEVSNMSR	10	DRB1_0401	27.9	0.90	SB
DTTIALDNSTFKASA	11	DRB1_0401	29.5	1.00	SB
DYATEVSNMSRAQIL	12	DRB1_0401	30.2	1.00	SB
TTIALDNSTFKASAT	13	DRB1_0401	33.7	1.20	SB
ADTTIALDNSTFKAS	14	DRB1_0401	33.9	1.20	SB
VHPFMLNNDVCVHFH	15	DRB1_0401	42.5	1.70	SB
GHNFKAQPDLAEAAT	16	DRB1_0401	44.9	1.80	SB
TVHPFMLNNDVCVHF	17	DRB1_0401	45.4	1.90	SB
RAQILQQAGTSVLAQ	1	DRB1_0101	8.3	1.90	SB

*SB strong binder

**Table 6:** Predicted B-cell linear epitopes of PLAC1-fliC

**No.**	**Start**	**End**	**Peptide**	**Number of residues**	**Score**
1	378	487	QQKYKVSDTAATVTGYADTTIALDNSTFKASATGLGG TDQKIDGDLKFDDTTGKYYAKVTVTGGTGKDGYY EVSVDKTNGEVTLAGGATSPLTGGLPATATEDVKNVQVA	110	0.815
2	76	90	IHYSSKGTPSKFVIP	15	0.787
3	14	51	MVTVHPFMLNNDVCVHFHELHLGLGCPPNHVQPHAYQF	38	0.778
4	1	6	QSPMTV	6	0.74
5	237	261	GLRINSAKDDAAGQAIANRFTANIK	25	0.726
6	173	194	LDISEDWSLHTDDMIGSMGSGG	22	0.708
7	679	696	SVLAQANQVPQNVLSLLR	18	0.69
8	151	168	HTQVPCHQAGAQEAQPLQ	18	0.662
9	513	523	SYTDNNGKTID	11	0.654
10	107	123	SMRVASKSRATAQKDEK	17	0.649
11	298	310	AVQSANSTNSQSD	13	0.637
12	196	222	GGSGGSGMAQVINTNSLSLLTQNNLNK	27	0.61
13	138	145	NCDCPPCV	8	0.593
14	603	615	ATTTENPLQKIDA	13	0.588
15	647	658	NLTSARSRIEDS	12	0.585
16	347	359	TIQVGANDGETID	13	0.581

**Table 7:** Predicted B-cell discontinuous epitopes of PLAC1-fliC

**No.**	**Residues**	**Number of residues**	**Score**
1	:A298, _:V299, _:Q300, _:S301, _:A302, _:N303, _:S304, _:T305, _:N306, _:S307, _:Q308, _:D310, _:V377, _:Q378, _:Q379, _:Y381, _:K382, _:V383, _:S384, _:D385, _:T386, _:A387, _:A388, _:T389, _:V390, _:T391, _:G392, _:Y393, _:A394, _:D395, _:T396, _:T397, _:I398, _:A399, _:L400, _:D401, _:N402, _:S403, _:T404, _:F405, _:K406, _:A407, _:S408, _:A409, _:T410, _:G411, _:L412, _:G413, _:G414, _:T415, _:D416, _:Q417, _:K418, _:I419, _:D420, _:G421, _:D422, _:L423, _:K424, _:F425, _:D426, _:D427, _:T428, _:T429, _:G430, _:K431, _:Y432, _:Y433, _:A434, _:K435, _:V436, _:T437, _:V438, _:T439, _:G440, _:G441, _:T442, _:G443, _:K444, _:D445, _:G446, _:Y447, _:Y448, _:E449, _:V450, _:S451, _:V452, _:D453, _:K454, _:T455, _:N456, _:G457, _:E458, _:V459, _:T460, _:L461, _:A462, _:G463, _:G464, _:A465, _:T466, _:S467, _:P468, _:L469, _:T470, _:G471, _:G472, _:L473, _:P474, _:A475, _:T476, _:A477, _:T478, _:E479, _:D480, _:V481, _:K482, _:N483, _:V484, _:Q485, _:V486, _:A487, _:S513, _:T515, _:D516, _:N517, _:N518, _:G519, _:K520, _:T521, _:I522, _:D523, _:D542	133	0.787
2	:Q1, _:S2, _:P3, _:M4, _:T5, _:V6, _:M14, _:V15, _:T16, _:V17, _:H18, _:P19, _:F20, _:M21, _:L22, _:N23, _:N24, _:D25, _:V26, _:C27, _:V28, _:H29, _:F30, _:H31, _:E32, _:L33, _:H34, _:L35, _:G36, _:L37, _:G38, _:C39, _:P40, _:P41, _:N42, _:H43, _:V44, _:Q45, _:P46, _:H47, _:A48, _:Y49, _:Q50, _:F51, _:V65, _:S66, _:Q67, _:D68, _:M69, _:I76, _:H77, _:Y78, _:S79, _:S80, _:K81, _:G82, _:T83, _:P84, _:S85, _:K86, _:F87, _:V88, _:I89, _:P90, _:Q97, _:K98, _:S99, _:T103, _:C106, _:S107, _:M108, _:R109, _:V110, _:A111, _:S112, _:K113, _:S114, _:R115, _:A116, _:T117, _:A118, _:Q119, _:K120, _:D121, _:E122, _:N138, _:C139, _:D140, _:C141, _:P142, _:P143, _:C144, _:V145, _:H151, _:T152, _:Q153, _:V154, _:P155, _:C156, _:H157, _:Q158, _:A159, _:G160, _:A161, _:Q162, _:E163, _:A164, _:Q165, _:P166, _:L167, _:Q168, _:L173, _:D174, _:I175, _:S176, _:E177, _:D178, _:W179, _:S180, _:L181, _:H182, _:T183, _:D184, _:D185, _:M186, _:I187, _:G188, _:S189, _:M190, _:G191, _:S192, _:G193, _:G194, _:S195, _:G196, _:G197, _:S198, _:G199, _:G200, _:S201, _:G202, _:M203, _:A204, _:Q205, _:V206, _:I207, _:N208, _:T209, _:N210, _:S211, _:L212, _:S213, _:L214, _:L215, _:Q217, _:N218, _:N219, _:N221, _:K222, _:S679, _:V680, _:L681, _:A682, _:Q683, _:A684, _:N685, _:Q686, _:V687, _:P688, _:Q689, _:N690, _:V691, _:L692, _:S693, _:L694, _:L695, _:R696	177	0.687
3	_:G237, _:L238, _:R239, _:I240, _:N241, _:S242, _:A243, _:K244, _:D245, _:D246, _:A247, _:A248, _:G249, _:Q250, _:A251, _:I252, _:A253, _:N254, _:R255, _:F256, _:T257, _:A258, _:N259, _:K261, _:T347, _:Q349, _:V350, _:G351, _:A352, _:N353, _:D354, _:G355, _:E356, _:T357, _:D359, _:T644, _:N647, _:L648, _:S650, _:A651, _:R652, _:S653, _:R654, _:I655, _:E656, _:D657, _:S658, _:Y660	48	0.667
4	_:G371, _:L372, _:L375, _:A569, _:A602, _:A603, _:T604, _:T605, _:T606, _:E607, _:N608, _:P609, _:L610, _:Q611, _:K612	15	0.500

**Table 8:** Predicted CTL epitopes of PLAC1-fliC

**Peptide rank**	**Start position**	**Sequence**	**Score (ANN/SVM)** ^*^
1	9	SIDWFMVTV	0.73/1.2411843
2	36	GLGCPPNHV	0.96/0.71105708
3	61	RAKAVSQDM	0.97/0.5147798
4	83	TPSKFVIPV	0.88/0.5965804
5	56	TECGIRAKA	0.85/0.5788711
6	87	FVIPVSCAA	0.94/0.37276101
7	70	VIYSTEIHY	0.82/0.4671223
8	22	LNNDVCVHF	0.57/0.59104808
9	119	QKDEKCYEV	0.95/0.14603929
10	6	VLCSIDWFM	0.46/0.53298459

^*^Cut-off score: 0.51 (ANN) and 0.36 (SVM); ANN, artificial neural network; SVM, support vector machine
